# Impact of Adverse Childhood Experiences on Resilience and School Success in Individuals With Autism Spectrum Disorder and Attention-Deficit Hyperactivity Disorder

**DOI:** 10.7759/cureus.31907

**Published:** 2022-11-26

**Authors:** Ngozi J Adaralegbe, Okelue E Okobi, Zainab T O. Omar, Esther Segun, Endurance O Evbayekha, Adesewa Abolurin, Emmanuel O Egberuare, Henrietta C Ezegbe, Adeoluwa Adegbosin, Adebola G Adedeji, Ebikiye G Angaye, Ijeoma C Izundu, Babatunde O Oyelade

**Affiliations:** 1 Allied Health Sciences, University of Connecticut, Waterbury, USA; 2 Family Medicine, Arizona State University, Tempe, USA; 3 Family Medicine, Lakeside Medical Center, Belle Glade, USA; 4 Pediatrics, Dubai Medical College for Girls, Dubai, ARE; 5 Pediatrics, School of Biological Sciences and Applied Chemistry, Seneca College, Toronto, CAN; 6 Internal Medicine, St. Luke's Hospital, St. Louis, USA; 7 General Practice, University of Lagos College of Medicine, Lagos, NGA; 8 Urology, Southern Alberta Institute of Urology, Calgary, CAN; 9 Internal Medicine, Simon Fraser University, Vancouver, CAN; 10 Psychiatry and Behavioral Sciences, Kharkiv National University, Kharkiv, UKR; 11 General Practice, Olabisi Onabanjo University, Ago-Iwoye, NGA; 12 Family Medicine, Diete-Koki Memorial Hospital, Opolo, Yenagoa, NGA; 13 Clinical Research, Markham Stouffville Hospital, Markham, CAN; 14 Cardiology, Emory University, Atlanta, USA

**Keywords:** resilience, autism spectrum disorder and emotion, adverse childhood experience, attention deficit hyperactivity disorder (adhd), autism spectrum disorder (asd)

## Abstract

Adolescents with emotional and behavioral disorders face known academic challenges and poor life outcomes. It was imperative to explore and find if the new diagnostic criterion for diagnosing autism profoundly affects educational outcomes and resilience in individuals diagnosed with co-occurring autism spectrum disorder (ASD) and attention-deficit hyperactivity disorder (ADHD). The literature is robust on the impact of adverse childhood experiences (ACEs) on educational outcomes and resilience in adolescents with no history of disability. Still, there remains a dearth of literature explaining, with no ambiguity, the complex relationships between ACEs and resilience, school engagement, and success in individuals with co-occurring ASD and ADHD. This study reviews the existing scholarships on the topic. The significance of this review is that it informs healthcare providers, rehabilitation counselors, and educators about the need for early identification of individuals with ASD and ADHD with a background in ACEs. This will enable interventions early enough to ensure they are more resilient and can obtain improved success in school-related and outside-school activities and eventually improved quality of life.

## Introduction and background

For decades, researches have focused on studying adverse childhood experiences (ACEs) and their short-term and long-term consequences in individuals with neurocognitive disabilities [1-4] and those without neurocognitive disabilities. At some point in life, most of these people will face traumatic events that can directly or indirectly impact their mental health and quality of life tremendously. Children tend to experience these traumatic events more, often leading to an increased rate of developing long-term complications later in life [3-7]. The impact of traumatic events can result in the development of mental health conditions like anxiety, depression, post-traumatic stress disorder, and substance use disorders [3,4]. Childhood traumatic events also impact the physical health of people who experience them. Several pieces of literature have documented the consequences of ACEs on physical health, including the development of chronic medical illnesses such as hypertension, diabetes mellitus, and migraines [5-10]. When poorly managed, these traumatic events can negatively affect the quality of life of the individuals who experience them. For school-aged children, affected individuals tend to underperform in school, work, and the community at large [5-10]. The fact that the consequences of ACEs are not just limited to the individual but the community is also a cause for concern. According to the Centers for Disease Control and Prevention (CDC), these effects can also be economic, social, psychological, or physical [7].

Individuals with autism spectrum disease (ASD) and attention-deficit hyperactivity disorder (ADHD) are not spared from the consequences of ACEs itemized above, especially the ACEs of parental separation or divorce, parental substance use, and parental mental illness [1-34]. If anything, these individuals are more predisposed to both increased rates of experiencing ACEs [1-8, 10-17, 21-33] and increased rates of developing lifelong consequences [9, 10]. A growing body of literature discusses the complications of ACEs in ASD and ADHD as individual diagnoses [6-12, 33-45]; however, the literature that examines child resilience in a child who has ADHD coexisting in a person with ASD remains scant. It remains critical to explore what lifelong issues ACEs predispose adolescents to and interventions to abate those issues and improve educational outcomes, among other life outcomes. This review presents the pertinent literature discussing resilience in individuals with ADHD coexisting with ASD (ADHD_ASD) exposed to ACEs. The resilience model provides a theoretical grounding to understand the possible interactions between child resilience and school success in adolescents with these neurocognitive disabilities. This model explains the relationships between this study's significant concepts: ACEs, resilience, school engagement, and success in the population of interest. Succinct discussions on existing knowledge regarding the development of resilience in children with a diagnosis of ASD_ADHD will be provided while highlighting the dearth of literature that focuses on the development of individual resilience and its impact on school success and overall participation in life activities.

Suppose individuals with co-occurring ASD and ADHD have a reduced quality of life and are more predisposed to less successful life outcomes when compared to individuals with no neurodevelopmental disability. This underscores the need to study the subject of child resilience in those with ASD and ADHD, especially given their background exposure to ACEs. Child resilience is such an important protective factor that can reduce their chances of developing other mental health challenges, thus increasing their chances of improved functioning, participation, and success in school and other communities. Just as ACEs have been rigorously studied over time, the concept of resilience has also been studied by researchers. This is no surprise, given the need to circumvent the effects of these negative traumatic experiences to alleviate or alienate some of the attended complications of ACEs. Resilience is not inherent but a process [36-42]. The American Psychological Association (APA) defines resilience as the ability of an individual to adapt well in the face of adversity, trauma, tragedy, threats, or even significant sources of stress [18]. The discussion of ACEs and resilience in this project is inadequate and incomplete unless the interaction of the positive protective and damaging risk factors that interact to determine one's ability to develop resilience is considered, especially in the population of interest in this review. This is where the resilience model is critical as the model captures the intricate connections between the positive protective factors and the negative risk factors and helps to determine an individual's quality-of-life outcomes [43, 44]. It suffices to state that resilience is not a binary concept, but the concept of resilience can be examined on a continuum as it is not a static variable. The environment determines how an individual develops resilience over time [36-45]. Being a strong protective factor, the ability to develop stable relationships cannot be overemphasized.

High resilience has been linked to improved participation and quality-of-life outcomes [2-10, 12-14]. The present study also explores the possible relationships between participation and success in life outcomes after exposure to ACEs in ASD_ADHD. This work fills a specific gap in the literature regarding developing individual resilience. This project is essential as it will help to investigate the relationships between exposure to ACEs, resilience, and school engagement and success. If these relationships are established and properly understood, then the factors that promote the development of resilience in the study population can also be explained. This presents opportunities to develop strength-based models that will emphasize the bolstering of strengths in adolescents with a co-occurring diagnosis of ASD and ADHD. A paradigm shift from understanding the complications of ACEs in individuals to researching methods that promote resilience, especially individual resilience, is essential. This is crucial at this time, given the increasing separation and divorce rates and substance use disorders, among other traumatic events that adolescents are exposed to. Besides, the need for a study of resilience in children, especially concerning their health (mental and physical), has been pointed out [1-13]. This study is thus critical and timely as it is needed to help fill this knowledge gap in the existing resilience literature.

The objective of the study

Review existing scholarships, and explore and delineate the relationship between ACEs and their impact on school success and the development of resilience in individuals with a diagnosis of ASD and ADHD. The primary goal of this study is to attempt to identify the factors that play a role in the development of resilience in adolescents with a co-occurring diagnosis of ASD and ADHD. This has never been done before. The overarching aim is to present an overview of existing literature while trying to bridge the existing gap in knowledge about ASD and ADHD as well as resilience and ACEs.

Method of literature search

**Figure 1:**
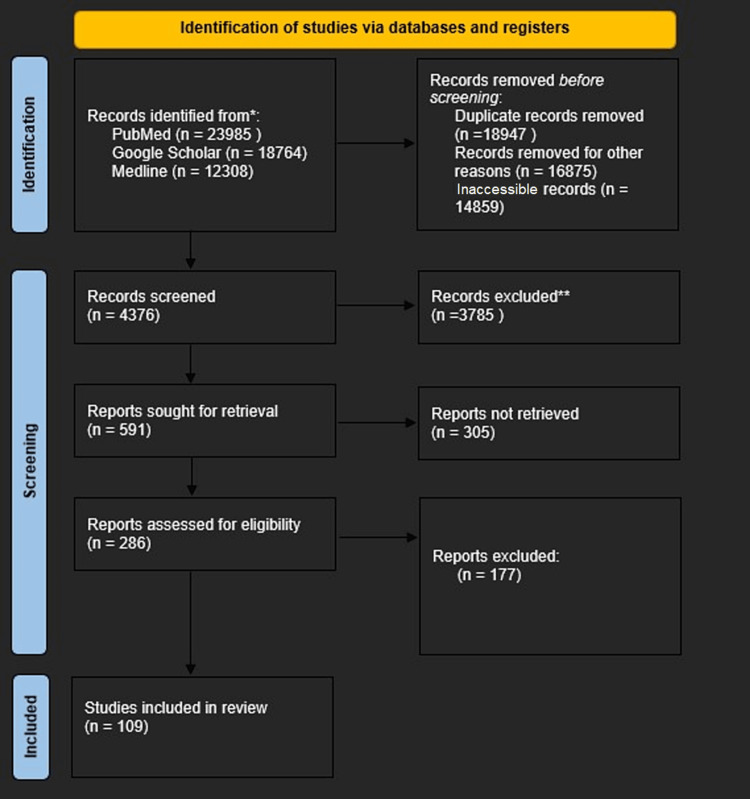
PRISMA flow diagram PRISMA: Preferred Reporting Items for Systematic Reviews and Meta-Analyses.

In this study, a thorough literature review was done to retrieve publications aligned with the study's aims and objectives. The literature search strategy in this study was done by accessing online databases, including PubMed, Google Scholar, and Medline. Specific keywords were used, which include 'resilience,' 'autism spectrum disorder,' 'attention-deficit hyperactivity disorder,' 'autism spectrum disorders attention-deficit hyperactivity disorder,' and 'school success,' 'school engagement,' 'adverse childhood experiences,' and the boolean operators of OR, AND, and NOT were also utilized in this literature search. The PRISMA (Preferred Reporting Items for Systematic Reviews and Meta-Analyses) flowchart in Figure 1 was created based on the study's established inclusion and exclusion criteria, which are listed in Table 1.

**Table 1:** Inclusion and exclusion criteria

Inclusion criteria	Exclusion criteria
1) Literature relevant to autism spectrum disorder, attention-deficit hyperactivity disorder, adverse childhood experience, and resilience.	1) The studies that did not discuss autism spectrum disorder, attention-deficit hyperactivity disorder, adverse childhood experience, and resilience were excluded because the objective of the study was focused on the interrelationships.
2) The studies must be original (randomized clinical trials, systematic reviews, meta-analyses) in which the interplay between autism spectrum disorder, attention-deficit hyperactivity disorder, adverse childhood experience, and resilience is discussed.	2) Opinion pieces and non-scholarly articles, secondary studies, scoping reviews, and research approaches other than primary studies were excluded.
3) Human studies.	3) Animal studies.
4) The studies must be published in a peer-reviewed journal to maintain validity and reliability of the studies.	4) The studies that were published in non-peer-reviewed journals and dissertations were excluded.
5) The studies must be originally published in English for readability by the reviewers.	5) The studies originally published in a language other than English were discarded.
6) Works of literature published between 1960 and 2022.	

## Review

Autism spectrum disorder (ASD)

ASD is a communication and behavior disorder prevalent worldwide, especially in the United States, and its incidence and prevalence rates continue to increase [7-14, 15-24]. According to some authors, the prevalence of ASD has risen dramatically over the years [7-18]. The scope of understanding of ASD has evolved over the years [7-15] and so have the etiology and the criteria for diagnosis. In the past century, when ASD was first coined, ASD was thought to be caused by the poor attachment of children to their mothers [7-16], which has since been further explored and unfounded [17-23]. Children with a diagnosis of ASD present with features that impact social interactions, speech development, and neurotypical behavioral development [15-18]. ASD is a communication and behavioral disorder; however, it is not without some associated clinical associations and comorbidities. Individuals with this diagnosis are also not exempted from the typical health conditions that affect other children without neurodevelopmental disabilities. Considering the social deficits and difficulty establishing social connections commonly associated with the diagnosis, this may affect their ability to cope with the effects of ACEs. Even without exposure to ACEs, ASD is associated with an increased rate of depression and less favorable social, academic, and health outcomes [13-19]. This study explores literature samples identifying any relationship between ACEs and resilience in the population of interest compared to people with a remote diagnosis of ASD or ADHD or adolescents without a neurodevelopmental disability. In addition to challenges with social communication and non-verbal communication behaviors, ASD is characterized by repetitive patterns in behaviors and routines [15-18]. This difficulty they experience in changing their interests and routines can be significantly affected by the social instability caused by parental separation or divorce [15-20]. According to Ahlers et al. (2017) [20], anxiety and social isolation are commonly associated with ASD, and this is often magnified with ACEs such as parental separation or divorce. Other authors also [15-21] documented significant marital strain in families with children who have emotional and behavioral disorders like ASD and ADHD.

Attention-deficit hyperactivity disorder (ADHD)

As ASD has evolved over the years, similar shifts have occurred for ADHD [22-33]. This neurodevelopmental disorder is characterized by inattention (attention deficit) and hyperactivity, with male children being predominantly diagnosed more than female children [22-26]. According to Barkley and Peters (2012), the diagnosis was first described by Melchoir Adam Weikard in 1775 [23]. The diagnostic criteria and terminology have also evolved, just like ASD. The labels used in the past were hyperkinetic reactions of childhood and attention-deficit disorder. With the introduction of the Diagnostic and Statistical Manual of Mental Disorders, Third Edition (DSM-III), the term ADHD was adopted [22-24, 26-33]. In DSM-IV, ADHD was grouped into three types: the ADHD-predominantly hyperactive-impulsive type, the ADHD-inattentive type, and the ADHD-combined type, which is a class for individuals who manifested both hyperactive-impulse symptoms and inattentive symptoms [25-36]. The evolution of this neurodevelopmental condition continued into the current DSM, the DSM-5, a most recent update to the manual [24-32]. Changes were made to the terminology used in describing the different types of ADHD; the age of onset was also modified to before age 12, and other changes in the presentation of the condition were not clarified in previous DSMs. ADHD is associated with diminished quality-of-life outcomes such as reduced academic success rates [26-27, 29-39], reduced employment [28, 29], and other health conditions [1-10, 17-25, 28-34]. The hyperactive-impulsive symptoms seen in individuals with ADHD have been reported to correlate with their increased school dropout rate and increased occupational impairment [30-39]. Some individuals with ADHD have an increased rate of being diagnosed with another mental health condition compared to the general population [18-30]. The mental health conditions associated with ADHD include anxiety, depression, obsessive-compulsive disorders, and opposition defiant disorder [31].

Autism spectrum disorder with attention-deficit hyperactivity disorder

While ASD is a diagnosis that commonly presents significant challenges with social skills and communication development [15-21, 32], ADHD, according to the name, has two major components. These two components are hyperactivity and impaired attention [21-32]. Just as the operational definitions and the general understanding have evolved over the years, so are the diagnostic criteria. Over the years, there have been some controversies in the criteria for diagnosing ADHD and ASD. A few years ago, when the DSM-IV was used, ADHD was regarded as a disorder occurring in isolation from ASD. This has, however, changed since the use of DSM-5 as ADHD can now be diagnosed in the context of ASD, given the overlapping features of both disorders. The ADHD-inattentive type is more common than the ADHD-combined type in individuals with ASD [21-33]. Whether ADHD is diagnosed in isolation or within the context of ASD, this diagnosis has been associated with an increased propensity to have less successful life outcomes and develop a mental health condition in the future [21-34]. Some authors, like Jang et al. (2013), also suggest that those with a dual diagnosis have a reduced level of functioning and, as such, are more predisposed to other psychological disorders [35]. Over the past few years, there has been an acknowledgment of the co-occurrence of ASD and ADHD. However, little is known about how one diagnosis significantly affects the impairments associated with the other [21-35]. A handful of studies have investigated other related areas. Authors like Goldin et al. (2013) [36] made a comparison of tantrum behavior profiles in children diagnosed with ASD and ADHD, while some other authors [21-37] recorded the effects of this dual diagnosis on reward anticipation. Other studies focused on structural brain abnormalities, and their findings illustrated that ASD and ADHD share similar brain abnormalities, structurally and functionally [31-38], not to mention that the two conditions also have a 50%-72% overlap of contributing genetic factors [39]. Additional research has demonstrated the need to further study these two populations as co-occurring disorders [21-33]. Gargaro et al. (2014) stated in their clinical study that children with co-occurring ASD and ADHD experience an increased rate of emotional and behavioral problems compared to those with a single diagnosis [40]. Another study that examined the quality of life in these individuals found that individuals with ASD and clinically significant ADHD were more likely to have less successful life outcomes than others with ASD or ADHD in isolation [41].

Autism spectrum disorder, attention-deficit hyperactivity disorder, and mental health

There is a need to consider the mental health of this population briefly. As stated earlier, the ability of an individual to excel in life depends significantly on their mental health functioning [46]. The parent's mental health is just as crucial as their children's since they affect one another. The ability of an adolescent to actively engage and succeed in school depends on their resilience and mental health status [47]. Research shows that children with ASD or ADHD are predisposed to developing an emotional or affective disorder as adults. The percentage for both affected populations could be as high [47]. This tendency to develop an emotional disorder like depression extends into the aging population with a similar diagnosis [48]. According to Nylander et al. (2013) [49], affective disorders were commonly seen in individuals with ADHD, while psychoses were common among individuals with ASD. Most adults with ADHD and ASD may have experienced some adverse childhood events. A pre-existing history of ACEs contributes more to their risk of having a mental health condition, given the primary diagnosis [2]. A parent with a mental health diagnosis stemming from whatever cause contributes significantly to the children's well-being.

Historical background of resilience

Human development is constantly being jeopardized because of events and adversities surrounding people in any given environment and at any given time. As highlighted earlier, these events have both short- and long-term consequences in the life of individuals. These consequences can trickle down to families and society at large. For example, many children are constantly affected by traumatic events within and outside their homes. United Nations Children's Emergency Fund reported that millions of children are displaced yearly because of conflicts and disasters. Millions of children are also being abused and neglected regularly, thus prompting the need to investigate factors that can help children develop resilience to escape the negative consequences that could arise from these exposures. These consequences include poor academic performance, less social engagement, and substance use disorders. To adequately understand the necessity of studying resilience in children, there is a need to briefly describe the historical events that propelled researchers to venture into resilience studies in children. Post-World War II (WW II), the field of rehabilitation was constituted to help returning veterans reintegrate into society; there was also a rapid shift of focus to finding out about the welfare of children exposed to war. According to Masten (2014) [50], many of these children were assessed by clinicians and were found to have varying levels of psychological dysfunction. Studies were carried out to assess the mental health of children exposed to WW II. Some factors were identified to be more associated with the development of mental health problems in the group studied. As more researchers developed an interest and delved into this area of research, they found that children exposed to wars and traumatic events had varied outcomes. According to Cicchetti (2013) [51], this led to even more studies to determine why some children fared better than others despite being exposed to the same events. Today, children continue to experience traumatic events in their lives, and as such, resilience in children should be studied, especially in those with highly stigmatized medical conditions like ASD/ADHD.

Resilience

The word resilience is of Latin origin and derived from the word "resilire," which means "to rebound." Resilience is a term that has gained so much attention among researchers and scholars. It is a term that has been used to promote research in at-risk groups, considering exposure to adversities [44-59]. There are different schools of thought on what exactly resilience is [41-45, 51-59]. While some literature has described it as a personal characteristic [45-52], in some other literature, it is described as not static [51-59]. However, a process that the environment, as such, can influence is an interaction between personal and environmental factors [53-59]. For a few others, resilience is an outcome that results despite adversities [54]. For example, Ungar (2008) [55] provided a more robust definition of resilience and described resilience as the capacity of individuals to navigate their way to health-sustaining resources, including opportunities to experience feelings of well-being and a condition of the individual family, community, and culture to provide these health resources and experiences in culturally meaningful ways.

Resilience theory was first postulated to explain the relationship between traumatic events and how these unpleasant life events impact individuals who experience them. Antonvsky in 1979 [55-91] sought to find out why people exposed to the same stress are impacted differently, with one group adversely affected and the other not succumbing to their negative experiences. This culminated in the generation of the resilience theory in 1979. Resilience is beyond coping and includes the ability to cope within and across systems and processes [42, 92]. From 1979 till now, many researchers have utilized this theory and conducted research that seeks to understand so many social and health issues. Examples of such studies include mental health outcomes secondary to adverse experiences [92-94]. Although resilience implies interactions of different systems, such as the individual, family, neighborhoods, and schools [82, 92-95], resiliency theory is best suitable for this study because it provides a strength-based focus on the issues of childhood traumatic events and will help inform interventions targeted at this stage of development [94]. Zimmerman identifies some positive factors, including individual, social, and contextual factors, and these positive protective factors help mitigate the effects of negative experiences. In this study, resilience is hypothesized to be a mitigating factor of mental health and school engagement or performances of adolescents with co-occurring ASD and ADHD who have experienced ACEs. These outlined models explain the relationships between ACEs and resilience. However, the attachment and resilience theories are suitable for explaining this current study's theoretical underpinnings.

Types of resilience

As stated earlier, the term resilience has been studied in some detail over the decades, and some types of resilience have been described by researchers based on their empirical findings [41-59]. Therefore, it is important to highlight these different types of resilience, so one can further appreciate the need to study individual resilience in adolescents with co-occurring diagnoses of ASD and ADHD, which is the scope of this study. Resilience has been classified into different categories in the literature, including individual, family, community, and even resilience-based policies. Some types of resilience that will be briefly described include family, community, and child (individual) [41-59]. However, one cannot overemphasize the fact that resilience is not an inborn quality [41-46, 51-56]. In agreement with the existing literature and as earlier highlighted, Egeland et al. in 2009 described resilience as a process rather than just an inherent ability [56]. Egeland et al. (2009) further stated that resilience develops over a period and is often because of person-environment interactions, so there is a need to explore the factors that may promote its development among adolescents [56, 57]. Since the concept of resilience has been studied by researchers, attempts have been made to understand it better and aid researchers in simplifying and narrowing the focus of their studies. Part of the attempt to properly understand the phenomenon of resilience led to the categorization of resilience. Cross-cultural perspectives on resilience and deployment resilience have also been described. According to Rak and Patterson in 1996 [58], resiliency in children is the capacity of those exposed to identifiable risk factors to overcome those risks and avoid negative outcomes such as delinquency and behavioral problems, psychological maladjustment, academic difficulties, and physical complications [58].

Family Resilience

Family resilience was described by McCubbin et al. in 1996 as the positive behavioral patterns and functional competence that individuals and the family unit demonstrate under stressful or adverse circumstances [59]. This determines the family's ability to recover by maintaining its integrity as a unit while ensuring, and where necessary restoring, the well-being of family members and the family unit as a whole. Families play a crucial role in the lives of children with special needs, such as adolescents with ASD/ADHD. Due to the increasing (emotional and financial) needs of families with children with ASD compared to families with children with no disabilities [59-65], family resilience is a crucial aspect of resilience to be considered. Concerning family resilience, Kahana et al. in 2015 [65] investigated family resilience among families with an individual diagnosed with ASD, and their findings revealed that resilient families were able to cope better with the adverse impact of quality-of-life stressors.

Individual Resilience

Individual resilience is the capacity for successful adaptation, positive functioning, or competence despite high-risk status, chronic stress, or following prolonged or severe trauma [56, 57]. Also, according to Kaplan (1996), resilience is primarily defined as the presence of protective factors (personal, social, familial, and institutional safety nets), which enable individuals to resist life stress [60]. An individual's resilience level can be measured by determining the ratio between protective factors and hazardous circumstances [60, 61]. Resilience in adolescents is a critical topic to investigate, and many pieces of literature currently exist that explore resilience in adults. While some research focused on exploring the development of resilience in adults concerning the development of chronic illnesses like diabetes mellitus and hypertension [61-63], others have explicitly focused on developing mental health conditions [64-69]. All these studies have been carried out in neurotypical individuals, thus creating a need to delve into resilience in adolescence, especially those with a dual diagnosis of ASD/ADHD, given the nature of their diagnoses and social interactions.

Community Resilience

Other than family resilience, community resilience is another concept that has been highlighted in pieces of literature [42-59]. While this is a vital concept and an important study to embark on, there is limited study on community resilience and ASD. While all these aspects of resilience are essential and should be studied, there is a need to adjust the focus of research from caregivers and the community to the individuals with the diagnosis. This will also find out possible factors that helped or can help them develop resilience even at the adolescent level of development.

Positive Protective Factors, Negative Risk Factors, and Resilience

**Figure 2:**
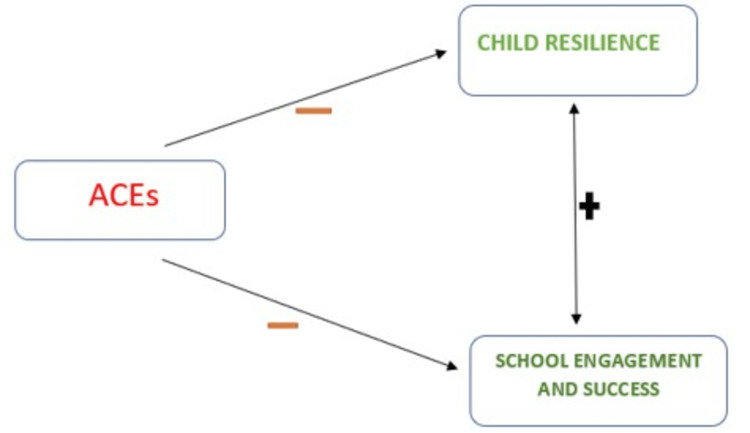
Relationship between ACEs, resilience, and school engagement and success Image credit: Dr. Ngozi Adaralegbe. ACEs: Adverse childhood experiences.

The concept of resilience is not a binary concept that can either be absent or present; it is a continuum [66-72]. Individuals can be said to have high or low resilience [9, 67] and can affect the quality of life and outcomes such as engagement and performance in school as illustrated in Figure 2.

The resilience of any individual also varies depending on the context being examined, whether it is resilience at home, in the school environment, or within the community. At the same time, greater resilience is associated with greater chances of positive mental health outcomes and improved quality of life [51-62, 64-71]. A lesser resilience in any individual is associated with reduced quality-of-life outcomes and the accompanying mental health conditions such as anxiety, depression, and PTSD [42-47, 68-71]. The concept of child resilience has been investigated, and some frameworks have been adapted to measure child resilience effectively. One such framework is the Health Outcomes from Positive Experiences (HOPE) framework, which includes concepts that buttress positive protective factors, activities, and community participation. The HOPE framework concepts, which exemplify the positive protective factors studied in the data set, have been previously used and validated [19, 70]. The concepts include emotional competencies, safe and stable neighborhoods, opportunities for social engagement in school and home, and nurturing relationships.

**Figure 3:**
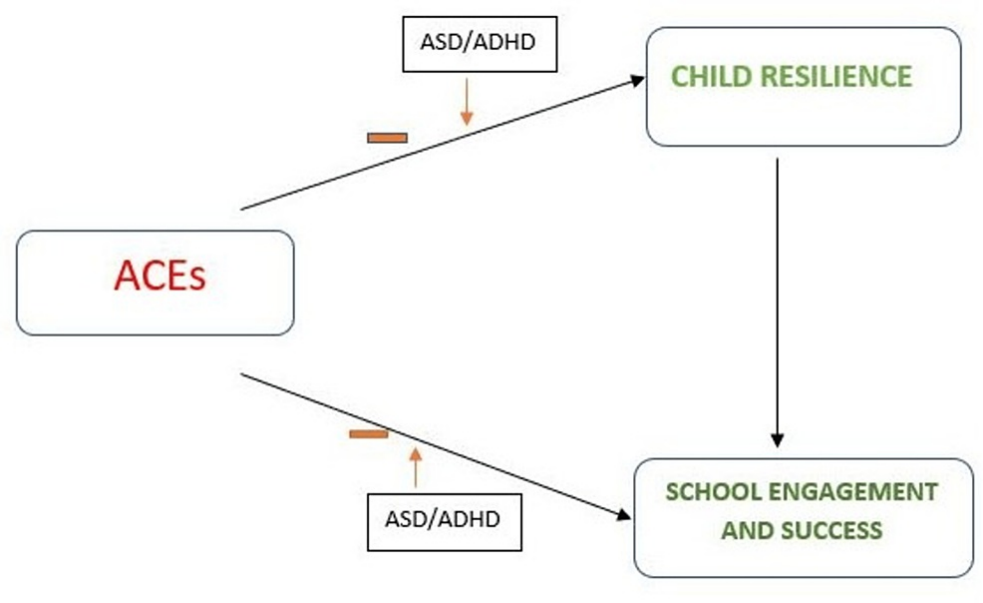
Possible impact of co-occurring ASD/ADHD on the outcomes of the study Image credit: Dr. Ngozi Adaralegbe. ACEs: Adverse childhood experiences; ASD: Autism spectrum disorder; ADHD: Attention-deficit hyperactivity disorder.

Similarly, as shown in Figures 2, 3, there is a negative correlation between these variants (resilience, ACE, and ADHD_ASD). Negative risk factors that can affect resilience, school engagement, success, and other quality-of-life outcomes are negative social interactions, genetics, economic hardship, parental separation or divorce, parental death, household incarceration, witnessing household violence, witnessing neighborhood violence, household substance use, household mental illnesses [19, 62, 70]. Conversely, positive protective factors that can preserve an adolescent against developing already highlighted complications include opportunities for social engagement within their school and home environment, nurturing relationships, and living in a safe neighborhood. Lastly, learning emotional competencies, the individual's activities, and community participation level are also considered. According to Barrett in 2020 [71], emotional and behavioral disorders and other mental illnesses impact adolescents and youths, thus presenting a need to study and explore these positive protective factors that can enhance the development of resilience in already stigmatized populations.

Resilience, ACEs, and mental health

When individuals continuously go through stressful conditions, especially over a prolonged period, there is neurobiochemical feedback, which results in the release of stress hormones in the body [1-8, 72-81]. In the long term, these hormones have been implicated in developing mental health conditions, namely anxiety, depression, and PTSD [72-84]. Developing resilience may alleviate the impact of adversities that individuals encounter daily, especially in their childhood [73-78, 81-100]. Resilience thus serves as a protective factor for mental health disorders, and the reverse is also true, and these changes can persist well into old age. Michielsen et al. in 2012 [48] reported depressive symptoms in older adults with ADHD. It is only logical to imagine that with an increased number of ACEs in an individual with a dual diagnosis of ASD_ADHD comes a more significant risk factor for a mental health disorder and, thus, the need to develop resilience.

Adverse childhood experiences (ACEs)

According to CDC (2020), adverse childhood events or ACEs are traumatic events that occur in an individual's life during a tender age of 0-17 years. Millions of children are constantly neglected [51]. These ACEs can be physical, psychological, emotional, or sexual traumatic events, including abuse, neglect, and household dysfunction [62-71, 92-100]. Similar to having a diagnosis of ASD or ADHD, or any other cognitive impairment, ACEs have been linked to the development of chronic mental and physical health conditions, even substance use disorders. Significant literature has documented an inverse relationship between ACEs and resilience. As ACEs increase, resilience decreases, which is the case for the general population [1-13, 42-59]. Some studies have demonstrated an inverse relationship between resilience and ACEs in the ASD population [1-13, 67-72, 81-95, 100]. According to Rigles in 2017 [13], in a study involving a population with a remote diagnosis of ASD, it was found that individuals with ASD were more likely to experience more ACEs, but resilience is not associated with ACEs. It remains undetermined if this inverse relationship can be applied to adolescents with a co-occurring diagnosis of ASD and ADHD. This negative relationship is also affected by the number of the ACE count and the severity of the ACEs using well-known categories, where four have been used as a cut point. ACE counts are categorized into two [1-13, 74, 92-108]. The first category is individuals with less than four ACEs, and the second category will be individuals with four or more ACEs [74]. However, there is a general consensus in these pieces of literature that the more the ACE counts, the more negative it seems to be with resilience.

Adverse childhood experiences and quality-of-life outcomes

These ACEs harm individuals' overall well-being and quality of life, affecting academic success, employment, marital outcomes, and other events. Studies have shown a high positive correlation between the number of ACEs and the possible adverse outcomes on well-being [3-4, 62, 81, 95-101]. ACEs have a dose-response relationship with many other health conditions and life outcomes [1-13, 42-59, 75, 82, 83, 91-100, 105], such as school engagement and success. For any individual to function effectively, there is a role of active participation in their community [1-13, 21-35, 76-84]. A known benefit of participation in one's immediate community is the establishment of social networks, which help build one's social capital and support system [65-77]. For the adolescent population, these relationships are essential within the school environment, home environment, or any other environment they operate in [60-79, 95-100]. Engagement within the school environment is critical since adolescents spend most of their time at school. According to Tavernor et al. (2013) [80], to adequately measure an adolescent's quality-of-life outcome, their activity, emotional well-being, and physical well-being must be considered. Adolescents enrolled in primary or secondary education spend an average of about 40% of their day interacting with their school environment [81-89]. This time is spent engaging with teachers, students, other educators, and non-academic staff within the school environment. Some students use this time to participate in extracurricular activities such as sports, clubs, and organizations within the school environment [82-95]. School engagement has been directly linked to improved academic success among adolescent students [42-59, 82-84, 91-94, 96-100]. This level of active engagement in the school environment is typical of adolescents without neurodevelopmental disabilities. However, research shows that children with ADHD or ASD do not participate as much as other students without neurodevelopmental disabilities [85-86], and individuals with ASD are more likely to be socially isolated in class and on the school playground.

Adolescents with a single diagnosis of ASD and ADHD are more predisposed to the effects of ACEs in early school and have less successful performances [81-84, 87-92, 97-103]. For those diagnosed with ADHD, the symptom of inattentiveness is directly linked to their poor academic performances [23-39, 88]. This is further worsened by exposure to ACEs, stemming from an unstable family environment. Some adolescents with ADHD have a poor attachment to their parents, which robs them of the positive protective factors they need to be more resilient and excel academically [23-27]. The relationship strains that those individuals with ADHD experience with their parents, teachers, and peers further set the stage for even more reduced performances in school compared to individuals without neurodevelopmental disabilities [89]. Adolescents with a background of ACEs have been noted to have poor performance in school, even without any neurodevelopmental disability [70] or any other form of prior mental health condition. It is noted in some literature that adolescents that demonstrate high resilience tend to engage and perform well in school activities [82, 90-100]. However, the reverse is true for adolescents with low resilience [81-90]. According to Bethell et al. (2016) [19], resilience is a mitigating factor between ACEs, school engagement, and school success. However, the literature to prove similar phenomena in adolescents with a co-occurring diagnosis of ASD and ADHD is lacking. Thus, a study like this one is needed to determine whether and to what extent resilience serves as a mitigating factor between exposure to ACEs and school performance as well as engagement.

Over the years, researchers have used several theories to explore and comprehend the relationship between ACEs and resilience in the general population, including the socio-ecological model [50-58], attachment theories, and life stress paradigm [91-96]. The ability for individuals to develop resilience is an interaction of multiple systems [1-13, 65-73, 81-100]. The socio-ecological theory emphasizes the fact that an individual's ability to develop resilience and adapt to traumatic situations is greatly influenced by systems around them, such as connections to family and other relationships in the community, as well as public policies [100-109]. According to some authors, protective factors are available at the individual, family, and community levels [72-80].

Although the socio-ecological theory has been used in the literature to provide an understanding of ACEs and life outcomes, the theoretical concept will be rooted in resilience theory in this current study. In rehabilitation and other related therapeutic professions, there is a focus on individual empowerment, which can be built or rebuilt through positive psychology. This means that an individual can rise above life experiences such as ACEs and live an improved quality of life despite the adverse experiences. The importance of positive protective factors, such as strong interpersonal relationships, cannot be stressed enough. The resilience theory is also vital to examine as it explains the relationship between the positive predictive and negative risk factors. Finally, the resilience theory helps to provide a holistic view of the interaction between ACEs, school engagement and success, and overall quality-of-life outcomes of any individual.​​​

Attachment, School Environment, Resilience, and School Success

Attachment is the close emotional bond an individual develops, while autonomy shifts the power to the learner. Achievement is completing increasingly complex tasks, and a relationship of reciprocity is referred to as altruism [92-95]. As highlighted earlier, when individuals can establish great developmental relationships, they can become more resilient and excel in life, even if they have a history of and exposure to traumatic events such as the ACEs in childhood. When individuals do not develop these essential elements and thus resilience, there is a tendency to have an individual who, rather than thriving in life, will end up with reactive coping strategies and immature defense mechanisms, which could culminate into psychological and physical dysfunctions. This ultimately results in poor life outcomes and chronic health conditions [96, 97].

Environmental factors can either impede or facilitate an individual's development of resilience, more so in adolescents. Environmental factors such as social support can play a crucial role in the life of adolescence and determine if the individual can succeed in the present daily activities and has the potential to predict the quality-of-life outcomes in the individual, given a background of ACEs. This concept can be applied to individuals without neurodevelopmental disabilities and individuals with neurodevelopmental disabilities. The environments pertinent to developing resilience in any adolescent include the family and school. When a child has adequate parental support, they tend to perform better in their life endeavors, whatever this might be. A study investigating students' academic achievement revealed that perceived parental support correlated highly and strongly with the performances of the students [29]. Another study explored the school environment and found that students with perceived peer and teacher support performed remarkably well academically [98]. These supports highlighted and other factors are necessary for developing resilience in any individual, especially adolescents.

Role of interventions in improving resilience and educational outcomes

While much focus is on resilience, the capacity to flourish despite difficult conditions [89], surprisingly little academic work has been done on resilience in clinical child disorders, including ASD coexisting with ADHD. However, resilient children are generally classified as displaying a diversified collection of behaviors, talents, and characteristics. These characteristics include things like participating in prosocial actions or finding resources to deal with obstacles and recover from setbacks.

The importance of interventions in improving life outcomes in individuals with emotional and behavioral outcomes cannot be overemphasized. The literature has examples of noteworthy interventions that have been used to enhance the outcomes of this study's population of focus [97-108]. For example, Pfiffner et al. (2013) [99] conducted a study where collaborative school-home behavioral intervention was implemented for ADHD. The intervention focused on classroom behaviors as well as social and independence skills, and a pre- and post-survey was collected. Significant improvements were noted in academic achievements and student engagement, among other life skills measured. Effective interventions for children with ASD also exist, and these are often targeted at different outcomes. While some interventions were effective, others yielded below-expected outcomes.

Mackay et al. (2017) conducted a study to help improve resilience and prevent depressive symptoms in children with ASD [100]. Many of these researchers hypothesized that the students' levels of resilience and perceived stress might be linked to how happy they are with their lives, but there is not much evidence to support these claims [81-90].

## Conclusions

There seems to be a consensus that people who have ASD coexisting with ADHD face challenges of ACEs just like every other person in society, and this has a negative impact on their resilience. When their basic needs are met, they are more likely to thrive and excel, resulting in a higher quality of life overall. Understanding the factors that impede and promote resilience, school engagement, and success can help inform the development of necessary interventions for this study population. Such projects will undoubtedly benefit the general adolescent population as well. This study was exploratory in nature and emphasized the importance of studying adolescents with ASD, ADHD, and co-occurring ASD and ADHD. More research is clearly needed to better understand and learn more about this relatively new diagnostic criterion of ASD and ADHD in the DSM-5 as this would allow all appropriate service providers to serve them better. This calls for a need to engage in continuous data gathering, which could pave the way for a deeper understanding of the study population and ways to serve them better and enhance their quality of life.

## Appendices

The primary author and co-authors performed the following roles:

1. **Ngozi J. Adaralegbe:** Conceptualization, cross-referencing, and fact-checking; formal analysis and interpretation of data; visualization; writing, reviewing, and editing the original draft.

2. **Okelue Edwards Okobi:** Supervision, oversight, leadership, correspondence; methodology, literature search, approvals, project administration, re-writing, re-formatting, editing, and chart conceptualization.

3. **Zainab T. O. Omar:** Resources, inclusion and exclusion analysis, visualization, writing post drafts, and acting as a medical content editor.

4.** Esther Segun:** Resources, software, validation, visualization, writing of original draft, reviewing, and editing.

5. **Endurance O. Evbayekha:** Conceptualization, validation, visualization, writing, draft editing, and semantics formator.

6. **Adesewa Abolurin:** Reviewing, proofreading, writing, and editing the draft.

7. **Emmanuel O. Egberuare:** Article mobilization, project administration, resources, and oversight.

8. **Henrietta C. Ezegbe: **Validation, preview-validation, review, editing, and paragraphing.

9. **Adeoluwa Adegbosin: Preview validation**, content fact-checking, literature search, and visualization.

10. **Adebola G. Adedeji:** Overview of draft, conceptualization, visualization, and editing of draft and main.

11. **Ebikiye G. Angaye:** Resources, fact-checking, validation, and correspondence.

12.** Ijeoma C. Izundu: **Review of writing, project administration, review of the draft, writing, editing, content review, and input.

13. **Babatunde O. Oyelade:** Resources, fact-checking, validation, and correspondence.
